# Chemoimmunotherapy with brentuximab vedotin combined with ifosfamide, gemcitabine, and vinorelbine is highly active in relapsed or refractory classical Hodgkin lymphoma

**DOI:** 10.1038/s41409-019-0454-z

**Published:** 2019-01-30

**Authors:** Khadega A. Abuelgasim, Mohsen Alzahrani, Yousef Alsharhan, Moataz Khairi, Mohammed Hommady, Giamal Gmati, Hind Salama, Osama Ali, Bader Alahmari, Emad M. Masuadi, Ahmed Alaskar, Ayman Alhejazi, Moussab Damlaj

**Affiliations:** 1 King Abdulla International Medical Research Center, Riyadh, Saudi Arabia; 2 King Abdulaziz Medical City, Department of Oncology, Riyadh, Saudi Arabia; 3 King Saud bin Abdulaziz University for Health Sciences, Riyadh, Saudi Arabia

**Keywords:** Translational research, Prognosis

Salvage therapy followed by autologous hematopoietic stem cell transplantation (HCT) can be curative in relapsed/refractory classical Hodgkin lymphoma (cHL) [1]. Complete response (CR) prior to HCT, particularly complete metabolic response (CMR) indicated by a negative positron emission tomography/computed tomography (PET/CT) scan, is highly prognostic of post-transplant outcome [2, 3]. Thus optimization of disease status prior to HCT is highly desirable. There is no gold standard salvage in R/R cHL prior to HCT, and the choice of regimen depends on the physicians’ experience and preference. Brentuximab vedotin (Bv) has demonstrated excellent activity in cHL. Recent reports have combined Bv with standard salvage or with PD-1 inhibitor therapy with higher responses than conventional chemotherapy alone [4–6]. Our aim from this analysis is to examine the efficacy of Bv incorporated within the gemcitabine salvage regimen ifosfamide, gemcitabine, and vinorelbine (IGEV-Bv).

After institutional review board (IRB) approval, patients’ ≥ 14 years of age with relapsed or refractory cHL who received IGEV-Bv at our institution between 2013 and 2017 were identified, and all records were retrospectively extracted. Patients were eligible if they had histologically proven evidence of disease and those who achieved a partial metabolic response (PMR) or better to salvage therapy proceeded to HCT [7]. Patients received IGEV as first or subsequent salvage (FS or SS), as previously described [8]. Bv was administered at a dose of 1.8 mg/kg body weight on day 1 of each 3-week IGEV course. A minimum of two cycles of salvage were administered to all patients, and those who did not attain at least a PMR status following two cycles of salvage were switched to an alternate non-cross resistant regimen.

All analyzed patients underwent PET/CT staging following one or two cycles of IGEV-Bv to assess response. All studies were performed on GE 710 discovery TF system. Standardized uptake value of the liver and mediastinum is noted, and update was classified per Deauville criteria as ≤ liver uptake or ≤ mediastinal blood pool [9]. Patients with uptake ≤ liver (i.e., Deauville 3) were deemed to have CMR. Patients received BEAM (carmustine, etoposide, cytarabine, and melphalan) as conditioning followed by autologous stem cell rescue. All patients were hospitalized during conditioning therapy and until platelet and neutrophil engraftment.

Overall survival (OS) was calculated from the date of stem cell infusion until the date of death of any cause or last documented follow-up. Progression-free survival (PFS) was calculated from the date of stem cell infusion until death of any cause or evidence of disease progression or relapse. Baseline patient, disease, and treatment-related variables were reported using descriptive statistics (counts, medians, and percentages). Probability of OS and PFS was computed using the Kaplan–Meier method. Group comparisons were made using the log-rank test. Statistical analyses were performed using JMP Pro Version 11 (SAS Institute, Cary, NC, USA) software and EZR on R commander.

A total of 28 patients met the eligibility criteria and were included in this analysis. The median age was 25 (15–49) years, and 15 (53%) were men. All patients had early unfavorable or advanced stage disease at diagnosis with 8 (29%) having a bulky mass and 20 (71%) with constitutional symptoms. A total of 12 (43%) patients had refractory disease with evidence of progression within 3 months following completion of front-line therapy. All patients achieved at least a PMR and were able to proceed to HCT. Response assessment after IGEV-Bv (one or two cycles) showed CMR in 20 (71%) and PMR in 7 (25%) and stable disease in 1 (4%). Baseline characteristics and response are shown in Table 1. The most common toxicities observed were hematologic with grades 3–4 neutropenia and thrombocytopenia at 96% and 89%. Median number of units of red blood cells transfused during the course of salvage therapy was 2. Febrile neutropenia was seen in 16 (57%). No transfer to the intensive care unit or mortality during salvage chemotherapy. Other observed adverse events (AE) are shown in Table 2.

**Table 1:** Baseline characteristics and therapy of the cohort

Characteristics	Entire cohort (*n*=28)
Male, *n* (%)	15 (53%)
Age at HCT, median (range)	25 (15–49)
Stage at Dx, *n* (%)	
II	7 (25)
III	6 (21)
IV	15 (54)
Constitutional Symptoms at Dx, *n* (%)	20 (71)
Bulky Disease at Dx, *n* (%)	8 (29)
Front Line Treatment, *n* (%)	
ABVD	23 (82)
ABVD → EscBEACOPP	4 (14)
Other	1 (4)
IFRT, *n* (%)	5 (18)
Refractory (≤ 3 months remission), *n* (%)	12 (43)
Time to relapse, median months (range)	7.9 (1.9–133)
IGEV-Bv order of salvage, *n* (%)	
First	14 (50)
Subsequent	14 (50)
No. of salvage cycles, median (range)	2 (2–6)
Number of cycles at stem cell collection, median (range)	2 (1–5)
Days of collection, median (range)	1 (1–2)
CD34x10^6^/kg collected, median (range)	13.6 (2.8–44.8)
PET/CT status post IGEV-Bv, *n* (%)	
Complete metabolic response	20 (71)
Partial metabolic response	7 (25)
Stable disease	1 (4)
Median follow-up, months (range)	17 (0–65)

*HCT* hematopoietic stem cell transplant, *Dx* diagnosis, *PET/CT* positron emission tomography/computed tomography, *ABVD* doxorubicin, bleomycin, vinblastine, and dacarbazine, *escBEACOPP* escalated bleomycin, etoposide, doxorubicin, cyclophosphamide, vincristine, procarbazine, and prednisone, *IFRT* involved field radiotherapy, *IGEV-Bv* ifosfamide, gemcitabine, and vinorelbine with brentuximab vedotin

**Table 2:** Observed adverse events following IGEV-Bv salvage and Bv consolidation

Adverse event	Entire cohort (*n* = 28)
Neutropenia grades 3–4, *n* (%)	27 (96)
Thrombocytopenia grades 3–4, *n* (%)	25 (89)
Blood transfusion units, median (range)	2 (0–11)
Mucositis, *n* (%)	
Grades 1–2	5 (18)
Grade 3	1 (4)
Febrile neutropenia, *n* (%)	16 (57)
Peripheral neuropathy on salvage, *n* (%)	
Grades 1–2	2 (7)
Grade 3	1 (4)
Diarrhea, *n* (%)	6 (21)
Transaminitis grades 3–4, *n* (%)	2 (7)
Acute renal injury, *n* (%)	0
ICU transfer, *n* (%)	0
Bv consolidation, *n* (%)	18 (64)
Indication for Bv consolidation, *n* (%)	
Remission < 12 Months	13 (72)
B-symptoms at Relapse	3 (17)
Extranodal Relapse	2 (11)
Total doses of Bv delivered, median (range)	15 (6–16)
Filgrastim given during Bv consolidation, *n* (%)	7 (39)
Peripheral neuropathy on consolidation, *n* (%)	
Grades 1–2	4 (22)
Grade 3	1 (5)
Bv dose reduced due to AE, *n* (%)	6 (33)

*HCT* hematopoietic stem cell transplant, *ICU* intensive care unit, *IGEV-Bv* ifosfamide, gemcitabine, and vinorelbine with brentuximab vedotin, *AE* adverse events

Median (range) of CD34 collected cells were 13.6 (2.8–44.8) × 10^6^/kg after a median of two cycles of salvage chemotherapy. Consolidative Bv post HCT was given to 18 (64%) mainly due to relapsed disease within 12 months following front-line therapy with a median (range) of 15 (6–16) doses administered pre- and post-HCT. Dose adjustment in Bv was done in 6 (33%) due to AE predominantly neutropenia or neuropathy as shown in Table 2.

**Fig. 1:**
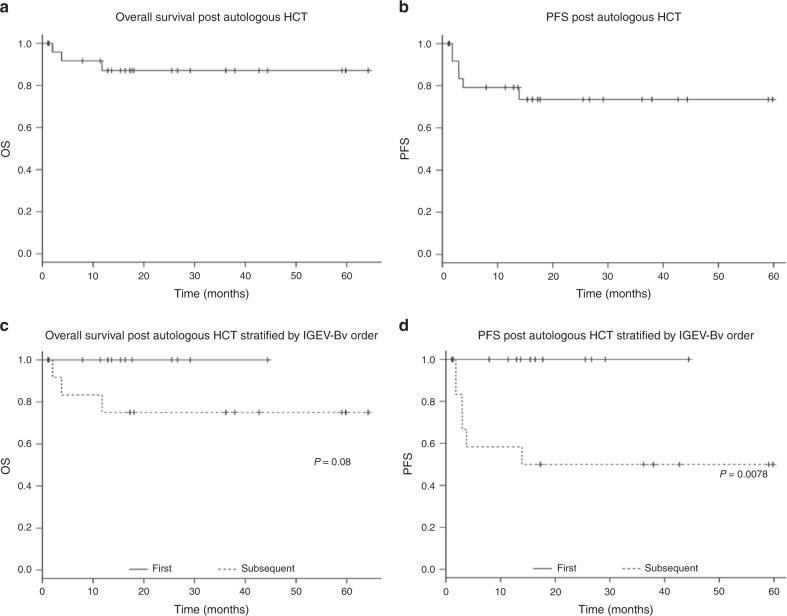
Progression-free survival (PFS) and overall survival (OS) post autologous HCT. **a** OS for the whole cohort; **b** PFS for the whole cohort; **c** Overall survival stratified by IGEV-Bv salvage order; **d** PFS stratified by IGEV-Bv salvage order

Post HCT, the estimated 2-year PFS and 2-year OS were 87.1% (65–95.7%) and 73.5% (49.8–87.3), respectively. A total of six patients experienced disease relapse post HCT and three patients died; due to progressive disease in two and pulmonary infection in one. IGEV-Bv as FS vs. SS resulted in a superior PFS and trend toward improved OS at 100 vs. 75% (40.8–91.2) *p* = 0.0078 and 100 vs. 50% (20.8–73.6) *p* = 0.08, respectively. These results are shown in Fig. 1.

Autologous HCT is a potentially curative therapy in R/R cHL with approximately half of patients achieving prolonged remissions following standard salvage therapy followed by HCT [10]. Importantly, CMR status pre-HCT indicated by a negative PET/CT has been shown to strongly correlate with a superior outcome by a number of groups [2, 3]. Thus, optimization of disease status prior to HCT leads to higher remission rates. In this study, we observed that addition of standard dose Bv to IGEV salvage was associated with a 70% CMR rate leading to favorable post-HCT outcome in this high-risk cohort of patients. Importantly, ~50% of the cohort received IGEV as SS after failing to achieve PMR with previous salvage and fared relatively favorably than expected. Previously, Villa et al. reported that patients requiring a second salvage to attain disease control prior to HCT have a poor outcome with an estimated 5-year PFS and 5-year OS of only 11 and 20%, respectively [11].

The choice of salvage regimen in R/R cHL patients eligible for HCT is unknown and clinical practice varies among centers. Although no prospective comparisons of salvage regimens in the setting of R/R cHL were done, they appear to be comparable with regards to efficacy. Santoro et al. treated 91 patients with R/R cHL with IGEV and observed a relatively high response rates with ORR and CR rates of 81.3 and 53.8%, respectively, with a low-toxicity profile and high-mobilizing potential of stem cells [8]. As the outcome of patients can be optimized with deeper responses prior to HCT, and that CR status is not achieved in the majority of cases, ongoing efforts to further enhance responses with available regimens are underway.

Chemo-immunotherapy approaches combining Bv with salvage regimens to overcome chemotherapy resistance has been under active investigation. O’Conner et al. reported an international, multicenter phase 1–2 of Bv in combination with bendamustine (Be) as an outpatient salvage regimen in R/R cHL [12]. Importantly, the recommended dose for the phase 2 of the trial was 1.8 mg/kg of Bv and 90 mg/m^2^ of Be every 3 weeks corresponding to the standard dose of either drug as single agents in clinical practice. However, the proportion of patients achieving CR remains lower than desired and a concern regarding stem cell mobilization particularly in elderly patients > 60 years was observed. Be–Bv combination was also recently reported by LaCasce et al. in a group of 55 patients observing a CR rate of 73.6% with excellent post-HCT outcome [5]. A total of 31 patients received Bv monotherapy following Be–Bv, among them 25 patients received it in the setting of post-HCT consolidation. After a median follow-up of 20.9 months, the estimated 2-year PFS was 69.8% for those who underwent HCT and 62.6% in non-HCT recipients. Other groups examined the incorporation of Bv within an ESHAP backbone (BRESHAP) in 27 R/R cHL patients in a phase I/II trial; CR was achieved in 16 out of 17 evaluable patients prior to HCT with no grade III or IV toxicity [4]. More recently, attempts at salvage therapy examined the combination of Bv along with nivolumab in R/R cHL with preliminary results showing an impressive ORR of 82% with a CR rate of 61% and only a minority of patients (< 10%) requiring systemic steroids for immune mediated adverse effects [6].

This analysis has some important limitations, particularly with regards to the sample size and retrospective design. Furthermore, similar to the study by LaCasce et al., some patients received Bv as consolidation monotherapy post HCT, which may further enhance the post-HCT outcome as previously shown in the AETHERA trial [13]. However, 71% of patients achieved CMR status pre-HCT, which is perhaps the most important predictor of outcome as shown by multiple groups. Additionally, 27/28 (96%) of patients were able to proceed to HCT following IGEV-Bv. The follow-up is relatively short, but in a cohort of patients where over 70% progressed or relapsed within 1 year following front-line therapy, the majority of events are expected to take place within this follow-up time frame in such high-risk patients as shown previously by other studies [10, 13]. In conclusion, we demonstrate that IGEV-Bv is associated with high response rates even in heavily pre-treated patients without compromising stem cell mobilization leading to HCT in the majority of cases. Given the limitations of this analysis, these observations warrant further examination.
